# Queen Charlotte's Hospital

**Published:** 1919-10-11

**Authors:** 


### The Hospital

The Workers' Newspaper of Administrative Medicine and Institutional Life, Administration, National Insurance and Health.

No. 1740, Vol. LXVII. SATURDAY, OCTOBER 11, 1919. PRICE TWOPENCE

### QUEEN CHARLOTTE'S HOSPITAL.

It is now three weeks since the initiation of a correspondence in The Hospital concerning the conditions under which pupil-midwives carry out their training at Queen Charlotte's Hospital; and it is also three weeks since, in an editorial comment on the first of these letters, and on the Secretary's reply to it, a demand was voiced for a small independent commission of inquiry into the allegations against the management of this lying-in hospital. Since then a good deal more testimony has been submitted to us, and through our correspondence columns to the institutional world. Some of the evidence is contradictory, but on the whole it is impossible to avoid the conclusion that it points to a reasonable case for the demand as it was originally made in The Hospital (September 20, 1919, p. 620). Up to the time of writing we have not heard of any steps being taken to establish such an inquiry, without which the managers of the departments in question can be neither satisfactorily cleared nor regarded as guilty of charges which amount to gross incompetence and neglect of duty.

There are several ways in which a commission likely to command general confidence can be set up for these investigations. The Governors of Queen Charlotte's Hospital can appeal to various public bodies interested to nominate each a member—say, for instance, the College of Nursing, the British Medical Association, King Edward's Hospital Fund, and the Ministry of Health—the members to elect a chairman. A small committee constituted in some such way as this would meet the case amply. If the Governors will not take such an initiative, the impetus must come from outside: both the College of Nursing and the King's Fund, especially the latter, have sufficient standing to make any effort on the part of either of them too influential for the Governors to resist it by refusing facilities for the inquiry. Failing some such action, it will become the plain duty of the Ministry of Health to set the wheels in motion—an alternative which we have placed last because all Governmental action spells delay, and is liable to become the sport of party faction, with which this case should have nothing to do.

The members of any such body of inquirers need to be quite impartial, biased neither for nor against any of the parties to the dispute, and having no possible or conceivable personal interest in its outcome. More than that, they should include at least one individual with training in the laws of evidence and of experience in weighing such evidence. Last week, for example, two letters appeared in our correspondence columns (The Hospital, October 4, 1919, p. 19) giving diametrically opposite views and statements of fact concerning conditions at Queen Charlotte's. That one which supports the administration begins with the same three and a-half identical lines as the only other letter we have received on the same side (The Hospital, September 27, p. 641); and this curious similarity is noticeable also in the concluding sentence of each of the two letters. This may be nothing but coincidence, or it may be that one writer has copied her predecessor; but a trained lawyer would probably pursue the matter a little further, to find out whether the letters were inspired from a common source.

The Commission should include also at least one woman—indeed, preferably two, one a nurse and one a qualified medical woman. And there should also be at least one expert in hospital administration. A body so instituted, and allowed complete freedom by the Queen Charlotte's authorities for the examination of witnesses, whose names should not be disclosed, ought, in our judgment, to be set up without further delay. It should be within the terms of reference to inquire not merely into the specific allegations brought forward in our columns, but also into such cognate subjects as the number of pupils accepted at any one time compared with the clinical material available for their instruction; the suitability of the buildings, especially the pupils' quarters, for the uses to which they are put; the system of rationing and housekeeping, together with the allocation of responsibility as between the steward's, matron's, and kitchen departments; and, in fact, the whole organisation from the Hospital Committee downwards.

It will be seen from the particulars quoted in another column (p. 25) that a great amelioration has already taken place. This is gratifying in itself to all well-wishers of Queen Charlotte's Hospital, but it does not absolve the managers from the duty of fixing responsibility for past errors and of seeking independent expert advice as to further possibilities of improvement. At the same time it demonstrates that deficiences did exist, and justifies still further the demand for searching investigation.

